# The Treatment of Typhoid Fever

**Published:** 1894-09

**Authors:** Elmer Lee

**Affiliations:** Chicago.


ARTICLE IV.

### THE TREATMENT OF TYPHOID FEVER.

BY ELMER LEE, A. M., M. D., CHICAGO.

Read before the *Chicago Medical Society*, March 5, 1894.

Recognition of the value of cleanliness represents the most practical discovery in treatment during the present generation, and, at the same time it constitutes one of the really great discoveries in the history of Medicine. The application of the principles of cleanliness more nearly meets the requirements of a real advance in curative medicine than all the other propositions known to the profession for the cure of disease.

The symptoms of Typhoid Fever are two well known by all to need particular mention; the question of burning interest is what to do to be saved. The disease is produced by drinking contaminated water, and its seat of development is situated in the intestinal canal. There is a poison there which, if it could be removed before it had become absorbed into the blood, life, and even health would be spared. Allowed to remain, the poison is drawn into the circulation, and very soon the whole body feels the depressing effect. Even at this time, if those remaining poisonous juices and germs which are contained in the bowels were either neutralized by suitable remedies, or washed entirely away by a stream of flowing water, the disease would be checked, the patient spared, and health restored.

Without waiting for development of the symptoms of Typhoid Fever the very first proposition is to make the patient surgically clean, which means the free and abundant use of water internally first, and externally afterwards. The bowels are drenched and cleansed by a copious douche of hot soapy water, made to pass into and out of the lower bowel, until the contents are cleared away and the returning water comes back as clear as before it entered. The relief to the sick person by following such ablution is a delight to the physician and of greatest comfort to the patient. It seems so reasonable, they will say, and in practice it is just as good as they say. Fears were formerly entertained by me, as they are to-day by some of my contemporaries, that something would be bursted by running a large volume of water into the bowels of persons sick with Typhoid Fever. No harm has ever been done, and neither is it likely to be so caused. Several hundred cases have been so deluged by me with large quantities of water and in no instance has the result failed to be beneficial. The fear of doing harm may be entirely and forever dismissed. That which is not well understood by any one, always seems inconvenient, or troublesome to perform. But a little practice makes easy the methods which a little while before appeared unpleasant, even hard.

The temperature of the water used for cleansing and washing the bowels, should always depend upon the temperature of the body. If there is high fever the water is more agreeable and useful to the patient when it is cool, viz.: 75 degrees F.; but if the patient is chilly, or has a low temperature, the water should be at blood heat, nearly 100 degrees F. During the first week of illness, the irrigation of the bowels should take place in the morning and again in the evening of each day. After this, one douche of water should be given each day until convalescence. The co-operation of the patient is readily accorded. The treatment takes hold of his reason, which lends both hope and help to the management of the case.

Bathing the body is performed at regular intervals and by such a system as may be convenient and suitable to the individual. The bathtub may be used when the patient is strong enough to be assisted to it, where otherwise, sponging with cold water is very refreshing; and useful to maintain strength and lower the heat of the body.

The most effective and lasting influence is secured by wrapping the patient in a wet sheet. Two blankets are spread on the bed, covered with a sheet wet with cold water. The patient is wrapped in the sheet, and then folded quickly and completely in the blankets. The time during which the sick one may remain in the wet pack is from one half to one hour, or even longer if he is comfortable. Bathing opens the pores of the skin, and through them the system discharges a part of the hurtful waste of the body. This bathing should be continued, several times daily during the disease and during convalescence.

The internal treatment is uncomplicated, safe and useful. The basis of it is cold water, and always plenty of it to drink. Water cools the body and assists to cleanse it of the poison which makes it sick. The elimination is carried on through the intestinal canal, through the kidneys, through the lungs, and by the skin. Let the sick have water, it can do no harm in any case; water only does good. What cruelty it was in fever cases, to keep water from them and what suffering it caused. A half tablespoonful of hydrozone* is added to each glass of water. It is the best and most simple remedy that can be given that is likely to be of benefit in helping to cure Typhoid Fever. Continued for a few days, it is then laid aside for a few days and Glycozone substituted in its place, both as a relief to the patient and for the beneficial effect of the remedy itself. And so on in this way the two remedies are alternated, which is found by me to be the best arrangement for administering these valuable antiseptics. The preparation, Glycozone, is chemically pure, redistilled Glycerine in which Ozone, or concentrated Oxygen, has been incorporated, and can be taken with as much freedom and safety as pure Glycerine. The Glycozone may be taken in doses of half a teaspoonful to a glass of water as often as water is taken during the day. When it is desired to allay nervousness and induce sleep at night, sulphate of Codeine is used, in doses of from one-half to one grain, by the mouth, or one-quarter to one-half grain by the hypodermic method. This remedy tranquilizes the nervous system and induces sleep, and should be administered at night.

The Typhoid Fever patient receives as food, whatever is simple, at regular intervals of four hours. Milk, simple, natural milk, is nourishment of the highest importance. One egg every day, or every other day, is alternated with a small teacup of fresh pressed juice from broiled steak or mutton. The egg is pleasant to take and more nutritious, when whipped till it is light and then stirred with a small glass of milk. For a simple and nourishing artificial food, malted milk is always good.

The juices of fruits are delicious to the typhoid Fever patient, and are not to be dismissed on the supposition that they are injnrious. It is always interesting to observe that, when the fever is broken, and convalescence is beginning that water in copious draughts is no longer easy for

---

***Hydrozone now takes the place of Peroxide of Hydrogen, the strength is double, the dose one half. See addenda.**

the patient to take. When the usual glass of water is handed back half drained, it is an encouraging sign of beginning restoration. For wholesome drinking, fresh lake water which has passed through a Pasteur porcelain filter is entirely reliable.

The simplicity of the foregoing plan meets every requirement, and saves nearly every case, unless there is some complication. It is my belief that doing more than this is doing less, and less than this which is so simple is not enough. The profession agrees that no kind of drug treatment is useful or curative in Typhoid Fever, indeed, one of these days, in my opinion, the statement will be considered applicable to other, if not all, cases of diseases of the bowels.

The plan proposed by me and practiced during a period of five years, consists, in review, of the following systematic management in Typhoid Fever:

Water used internally as a douche for free irrigation of the bowels, either simple or made soapy with pure liquid soap. Water as a drink, and as a remedy taken copiously and frequently, especially during the stage of fever. Water s indispensable, and should be given as often as is desirable and agreeable to the circumstances of the case. Frequent application of cool water to the surface ot the body during the entire illness.

Remedies: Hydrozone and Glycozone for the antiseptic effect of the oxygen which is set free in the stomach and intestines. But to be of real value, these remedies are to be taken in considerable quantity largely diluted with water, else, in my opinion, they are of little use. The capacity of the bowels is so great that a little of anything cannot spread over enough of this enormous area to effect it beneficially. Cleanliness is the principle governing the use of Hydrozone and Glycozone.

For a remedy that sooths and brings on sleep at night sulphate of Codeine is better than chloral, besides it is the safest and best.

For food, anything that is simple and in liquid form; milk is always the best; milk and whipped eggs; pressed juice from broiled meat. The juice from fresh, ripe fruit. The nutrition taken should be at regular intervals (four hours), that sufficient time may be allowed for digestion.

Stimulants and drugs are injurious without exception, and better results are secured without their use. Typhoid Fever generally transmitted through the drinking water, is a preventable disease. Typhoid Fever affects all classes, but if food and water were always pure, no class or age need contract Typhoid Fever. Cleanliness everywhere and always is the means at hand which makes it possible to escape Typhoid Fever and other diseases of the bowels. Internal cleanliness as well as external is a reasonable proposition of hope for the cure of the unhappy multitude of sick and discouraged humanity.

103 State Street.

---

"The use of peroxide of Hydrogen as an internal remedy has been widely opposed by some of my patients, owing to the disagreeable metallic taste. This objection was partly obviated by the use of large dilution with water, but still not to my entire satisfaction.

Since reading the foregoing paper, a new antiseptic remedy called 'Hydrozone' has been received and examined already sufficiently, to promise relief from the objections against Peroxide of Hydrogen for internal use. Hydrozone has now been substituted by me instead of Peroxide of Hydrogen.

First, on account of its greater bactericide power, as it requires but half the quantity of the hydrozone to obtain the same result, and secondly, the taste of this remedy is not disagreeable to the patient."—*The Chicago Medical Recorder.*

